# Remote Photoplethysmography Is an Accurate Method to Remotely Measure Respiratory Rate: A Hospital-Based Trial

**DOI:** 10.3390/jcm11133647

**Published:** 2022-06-24

**Authors:** Edem Allado, Mathias Poussel, Justine Renno, Anthony Moussu, Oriane Hily, Margaux Temperelli, Eliane Albuisson, Bruno Chenuel

**Affiliations:** 1 CHRU-Nancy, Exploration Fonctionnelle Respiratoire—Centre Universitaire de Médecine du Sport et Activités Physiques Adaptées, F-54000 Nancy, France; m.poussel@chru-nancy.fr (M.P.); a.moussu@chru-nancy.fr (A.M.); o.hily@chru-nancy.fr (O.H.); m.temperelli@chru-nancy.fr (M.T.); b.chenuel@chru-nancy.fr (B.C.); 2 DevAH, Université de Lorraine, F-54000 Nancy, France; 3 OMEOS, F-54000 Nancy, France; justine.renno@omeos.co; 4 CHRU-Nancy, Direction de la Recherche Clinique et de l’Innovation, F-54000 Nancy, France; e.albuisson@chru-nancy.fr; 5 CNRS, IECL, Université de Lorraine, F-54000 Nancy, France; 6 Département du Grand Est de Recherche en Soins Primaires (DEGERESP), Université de Lorraine, F-54000 Nancy, France

**Keywords:** respiratory rate, Remote photoplethysmography, vital signs

## Abstract

Remote photoplethysmography imaging (rPPG) is a new solution proposed to measure vital signs, such as respiratory rate (RR) in teleconsultation, by using a webcam. The results, presented here, aim at evaluating the accuracy of such remote measurement methods, compared with existing measurement methods, in a real-life clinical setting. For each patient, measurement of RR, using the standard system (control), has been carried out concomitantly with the experimental system. A 60-s time frame was used for the measurements made by our rPPG system. Age, gender, BMI, and skin phototype were collected. We performed the intraclass correlation coefficient and Bland–Altman plot to analyze the accuracy and precision of the rPPG algorithm readings. Measurements of RR, using the two methods, have been realized on 963 patients. Comparison of the two techniques showed excellent agreement (96.0%), with most of the patients (n = 924—standard patients) being in the confidence interval of 95% in Bland–Altman plotting. There were no significant differences between standard patients and outlier patients for demographic and clinical characteristics. This study indicates a good agreement between the rPPG system and the control, thus allowing clinical use of this remote assessment of the respiratory rate.

## 1. Introduction

The modifications of vital signs, such as respiratory rate (RR), heart rate (HR), blood pressure, and oxygen saturation, have been identified as early warning factors of a patient needing intensive care in the case of dyspnea [1,2,3,4,5]. Although the recent rise of telemedicine offers several benefits, including enhanced care and improved time responses, it should also provide access to vital sign monitoring.

The RR is an essential physiologic variable for the early recognition of deteriorating patients. The average RR, in healthy adults at rest, stands between 12 and 18 breaths per minute. An RR under 10 or over 20 cycles per minute, while resting, is considered abnormal [6]. However, RR is often overlooked. Indeed, despite the necessity of monitoring patients on acute hospital wards at least daily, RR remains under-recognized and under-recorded [7,8]. According to a recent study, most nurses do not currently measure RR, and thus, they may overlook potentially ominous conditions [9]. In addition, Drummond et al. have highlighted a large interobserver variation, with measurement errors ranging from 2 to 6 breaths/min with video assessment [10]. This was also observed in the case of a video evaluation carried out by health professionals [11].

Other methods of evaluating the RR exist [12,13], but most are cumbersome and not so well-tolerated. Indeed, some systems are based on a measurement of the flowrate at the mouth and, then, require the use of a facemask that is not well-adapted for in-field monitoring [13]. Most RR measurement systems use signals from the thoracic and/or abdominal movement through strain sensors embedded into strap/chest belt or clothes [14,15,16]. However, these devices are not comfortable for the patient, and they are difficult to use in current practice for continuous or periodic monitoring. Therefore, a method able to precisely measure vital signs in a remote fashion, in a way that is both comfortable and accurate, would be a very valuable tool.

The photoplethysmography (PPG) technique, first described in 1937, is a simple, low-cost, and non-invasive optical technique used to measure the blood volume changes associated with the cardiovascular system [17]. In 2000, Wu, T et al. introduced remote PPG (rPPG), or the possibility of applying the PPG method in a remote fashion, to access vital signs from a distance [18]. Other studies suggested that vital health parameters could be measured from video recordings of one’s face under ambient light [19,20,21]. Remote PPG, using a conventional low-cost consumer’s webcam or a smartphone, is of particular interest since it is non-invasive, easy to perform, and would fit very well with the sharp rise of telemedicine use [21,22,23,24,25]. Several studies have used rPPG for measuring vital signs in laboratory settings in groups of up to 1328 subjects [24]. However, very few studies have been carried out on clinical settings, and even fewer have been on such a large sample of patients or, mainly, in the case of heart rate, as mentioned by Pham et al. [26,27,28,29].

To investigate the accuracy and reproducibility of the remote PPG method (rPPG) and show its efficiency in measuring RR (cpm) in clinical settings, we launched the first clinical trial on such a large and diverse cohort of patients that we also allowed the evaluation of its robustness to human variation (gender, age, BMI, etc.).

## 2. Materials and Methods

This interventional monocentric study was performed at a French hospital (University Hospital of Nancy) between December 2020 and May 2021. There were 1045 adult patients, managed in the Respiratory Function Exploration and Sports Medicine Department, who required a pulmonary function test and were included in the study. Patients aged over 18 years, with the ability to perform a pulmonary function test and with a stable clinical status, were involved in the study. The exclusion criteria were pregnant women or women of childbearing potential without effective contraception. The complete protocol and methods have been previously described [30].

Patients underwent a specific physical examination to collect gender, age, BMI, Fitzpatrick skin phototype (FSP), and resting RR [31]. Patients were at rest and comfortably seated on a chair in front of a computer using a webcam and rPPG system. RR (unit: cycles per minute (cpm)) measurements, using the experimental system rPPGc (Remote photoplethysmography imaging from I-Virtual, Caducy V1.0.0) and the standard acquisition system (control), were performed simultaneously. Uncompressed Videos were acquired at 30 FPS with the webcam of an ASUS laptop model: X512J, Intel^®^ I5 1.00 Ghz. Patients were seated at about 70–100 cm away from the camera. Measurements were performed in ambient light and natural conditions. PPG signals were extracted from the forehead region and chest movement. The methodology for measuring breathing rate falls within the domain of image processing. A thoracic breath has the effect of modifying the geometry of the rib cage, which causes a visible heaving on a 2D image. Changes in cage volume create contrast information in the image. Any pattern or shadow that sees its position modified over time is visible on a sequence of consecutive images. A specific processing of the image and the signals associated with each pixel makes it possible to follow the evolution of the variations, which correspond to the rhythm of breathing. Thus, the images that we collect for our measurements consist of the face part, which allows us to define the heart rate, as well as the lower part, which has an equivalent dimension under the face, allowing us to recover the variations of any sign either real, made up of patterns (ex: stripes), or induced by tissue folds (shadow). The processing of the collected signal is such that any frequency outside the 6 to 48 cpm zone is rejected. The control system is a chest belt (TN1132/ST Respiratory Belt ADInstruments). The TN1132/ST is used to observe respiration by measuring changes in abdominal or thoracic circumference. It contains a capacitive sensing element and custom electronics that respond linearly to length variations.

Readings and recordings were taken at three different time frames: 30, 60, and 120 s. The respiratory rate was assessed by counting the number of breathing cycles on different time bases during recordings of 120 s. The first 30 s were considered for the so-called 30 s recordings, and the first 60 s were considered for the so-called 60 s.

To analyze RR, descriptive analyses were conducted according to the nature and the distribution of the variable. Qualitative variables were described with frequencies and percentages; quantitative variables were reported as mean ± standard deviation (SD). The intraclass correlation coefficient (ICC), with a 95% confidence interval (CI), was used to measure the correlation between the two measurement systems. For interpreting ICC, values lower than 0·5, between 0·5 and 0·75, between 0·75 and 0·9, and greater than 0·90 were used as being, respectively, indicative of poor, moderate, good, and excellent reliability [32]. A Bland–Altman plot was applied, to describe the accuracy between the two measurement systems, using a 60-s time frame (rPPGc and control). The chi-square test or Fisher’s exact test with, if necessary, the exact calculation of Fisher, was used for the ordinal or nominal data analysis. We used the Student’s t-test to compare age and BMI. Binary logistic regression analyses were performed to study the association between patient status in Bland–Altman Plot and demographic variables (Standard Patients [in 95% CI means of difference] or Outlier Patients [out 95% CI means of difference]). We further analyzed the outlier patients for highlighting the cause of a measurement error. Analyses were performed using IBM SPSS Statistics V.23, and *p* values <0.05 were considered statistically significant.

This study has received approval from the French Ethics Committee (CPP TOURS-Région Centre-Ouest 1-2020T1-30 DM at 27 October 2020) and the French Agency for the Safety of Health Products (ANSM registration no. I-RCB 2020-A02428-31). It was conducted according to the European Good Clinical Practice (GCP) recommendations, the general ethical principles of the Declaration of Helsinki, and specific French regulations. Before inclusion, each patient received, both verbally and in written form, a full brief on the study objectives, its progress, and its constraints, before giving their written consent to participate in the trial. The protocol was registered at http://www.clinicaltrials.gov (ClinicalTrials.gov ID: NCT04660318). 

## 3. Results

A total of 1046 patients were eligible for the study, out of which 963 complied with the entire flow chart. Among them, five patients were excluded for non-consent and/or incomplete sets of measurements. Additionally, 78 patients were excluded for anomalies of the control acquisition system, as observed by the physician. In fact, we spotted connection issues due to a poor apposition of the band and highlighted the fact that a thoracic band mainly records upper chest breathing, while ventral breathing might be missed. The characteristics of the 963 tested patients are described in Table 1.

The correlation analysis between the experimental system and the control showed an ICC of 0.784 95% CI [0.672:0.975], indicating a good reliability.

**Figure 1:**
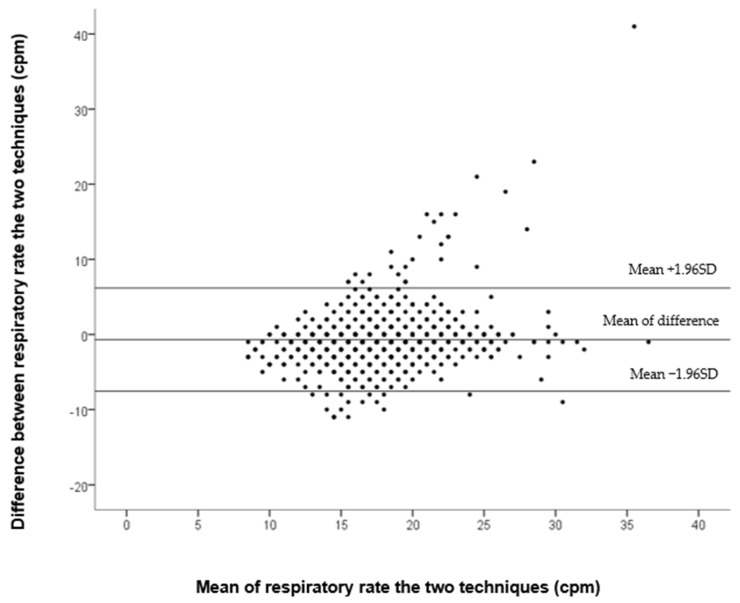
Bland–Altman plot showing the agreement between rPPGc et le control at 60 s.

Plotting of the data in the Bland–Altman plot showed 924 (95.9%) patients in the 95% confidence interval (Standard Patients) and, therefore, confirms the reliability of the system (Figure 1). The mean difference was 0.7 cpm and 95% CI [−7.6:6.2] cpm. We observed 39 measurements falling out of this range (Outlier Patients); further analysis pointed at 21 (2.2%) measurement errors originating from the rPPGc system compared to the control, which was the source of 18 (1.9%) errors.

There were no significant differences between Standard and Outlier patients for demographic and clinical characteristics. Further analysis showed that measurement errors didn’t originate from a given gender, age, BMI, or skin phototype. (Table 2). Those results were confirmed by the multivariate analysis shown in the Supplementary Table S1.

All groups considered, the Intra-system correlation between the experimental system, as a function of time, was substantial for measurements taken at 30, 60, and 120 s (Table S1).

## 4. Discussion

The study described good reliability of the RR measurements, between the control and the rPPGc techniques, with excellent agreement using the Bland–Altman plotting of the two systems. The correlation analysis between patients of the same group was reliable.

To our knowledge, this study is one of the rare studies to include patients in hospital conditions, with such a large population, and whose characteristics are well-described. The patients requiring pulmonary function tests are representative of the population of patients who would strongly beneficiate from accurate teleconsultation because, outside of the hospitalized patients, many are outpatients. The study was carried out on a larger population of 1328 patients, which aimed to analyze blood pressure in rPPG, and did not present a complete description of the population [24]. A few studies have already identified the skin tone as a potential limitation of rPPG use, with the main possible reason being a less favorable signal-to-noise ratio in darker skin types [33,34]. In our study, we counted 13 patients with an FPS greater than 5, so there was no evidence of outlier patients in this subgroup. The measurement of rPPG is based on an evaluation of blood flow at the subcutaneous level, and the effect of adipocyte can potentially be responsible for a measurement error. This was not demonstrated in our study. In fact, no effect of BMI was demonstrated. Looking at the literature, we did not find a study analyzing the effect of BMI with rPPG.

The level of correlation highlighted in this study is lower than that found in the previous study, where it is the lowest at 0.86, but on a population of less than 20 healthy subjects at most [35,36]. This difference can probably be explained by the fact that our study was carried out in a hospital, in current practice with non-specialist staff, unlike the studies which were in optimal laboratory conditions.

In our study, we noticed measurement errors of ±6.9 cpm and 95% CI [−7.6:6.2] cpm in Bland–Altman plotting. This result is twice as high as those observed in a previous study under laboratory conditions [36]. Therefore, the error range of our system is of the same range as the error observed between health-professionals [10].

Finally, this study highlights an excellent intrasystem correlation between the measurements of 30, 60, and 120 s. A measurement of 30 s seems sufficient for the evaluation of the RR in clinical conditions, which is consistent with the results of a previous study, which had carried out measurements over 20 s with excellent coordination [37].

The limitation of this study lies in two major points, with the first being the low number of FSP 5 and 6 patients, which is an issue that will be addressed in the near future. The second point to consider is that we measured RR, exclusively, in non-ambulatory and static environments. It would be interesting to investigate its accuracy in a mobile setting, where the RR increases in mean and in variability, as in exercise conditions.

The strength of the study lies in the fact that it is the very first to assess the applicability of the rPPG technique, in current practice, in such a large and diverse group of patients. Additionally, the tested population is representative of the patients who would benefit most from the use of such measurement tools in telemedicine, as it included all demographic characteristics (age, gender, BMI, phototype, in and outpatients of a large hospital).

## 5. Conclusions

Our study described good reliability of the RR measurements between the control and the rPPGc techniques, with excellent agreement using Bland–Altman plotting of the two systems, in the 6 to 48 cpm range. The correlation analysis between patients of the same group was substantial. To our knowledge, this study provides the largest series conducted in patients during real-life clinical settings, and it is the first to evaluate the accuracy of the rPPG assessment of RR in non-healthy subjects. Our results pave the way for a greater use of RR in current practice and broaden its measurement by non-experts or in telemedicine. However, clinical investigations will be needed to specifically address the limitations of this measurement technique in the case of specific contexts or diseases.

## Figures and Tables

**Table 1:** Baseline demographic and clinical characteristics of included patients (n = 963).

Female, n (%)	471 (48.9%)
Age, mean (SD), years	56.6 (±16.0)
Body mass index, mean (SD), kg/m^2^	28.1 (±7.3)
BMI < 30, n (%)	650 (67.5%)
Class 1 obesity, n (%)	172 (17.9%)
Class 2 obesity, n (%)	67 (7.0%)
Class 3 obesity, n (%)	74 (7.7%)
Fitzpatrick skin Color scale, n (%)	
1	20 (2.1%)
2	512 (3.2%)
3	360 (37.4%)
4	58 (6.0%)
5	8 (0.8%)
6	5 (0.5%)

Legend: Data are presented as n (%) for dichotomous variables, mean (±SD) for continuous demographic variables with normal distribution and median [interquartile range] with non-normal distribution.

**Table 2:** Demographic and clinical characteristics of ‘standard’ Patients and ‘Outlier’ Patients (according to the 95% IC Bland–Altman plot).

	Standard Patients (n = 924)	Outlier Patients (n = 21)	*p*-Value *
Female, n (%)	450 (48.7%)	11 (52.4%)	0.739
Age, mean (SD), years	56.5 (±15.9)	60.3 (±15.5)	0.278
18–29 years	72 (7.8%)	2 (9.5%)	0.221
30–39 years	85 (9.2%)	1 (4.8%)	
40–49 years	126 (13.6%)	0 (0.0%)	
50–59 years	193 (20.9%)	4 (19.0%)	
60–69 years	245 (26.5%)	7 (33.3%)	
70–79 years	145 (16.7%)	7 (33.3%)	
>80 years	49 (5.3%)	0 (0.0%)	
Body mass index, mean (SD), kg/m^2^	28.1 (±7.1)	28.5 (±7.4)	0.805
BMI < 30	628 (68.0%)	12 (57.1%)	0.444
Class 1 obesity	165 (17.9%)	5 (23.8%)	
Class 2 obesity	62 (6.7%)	3 (14.3%)	
Class 3 obesity	69 (7.5%)	1 (4.8%)	
Fitzpatrick skin color scale			
1	18 (1.9%)	0 (0.0%)	0.975
2	492 (53.2%)	12 (57.1%)	
3	344 (37.2%)	8 (38.1%)	
4	57 (6.2%)	1 (4.8%)	
5	8 (0.9%)	0 (0.0%)	
6	5 (0.5%)	0 (0.0%)	

Legend: Data are presented as n (x%) for dichotomous variables, mean (±SD) for continuous demographic variables with normal distribution and median [interquartile range] with non-normal distribution. * The chi-square test or Fisher’s exact test with, if necessary, the exact calculation of Fisher, was used for the ordinal or nominal data analysis. We used the Student’s t-test to compare age and BMI.

## Data Availability

Not applicable.

## Supplementary Materials

The following supporting information can be downloaded at: https://www.mdpi.com/article/10.3390/jcm11133647/s1, Table S1: Intra-system correlation between rPPGc as a function of time.

## References

[1] Goldhill D.R., McNarry A.F., Mandersloot G., McGinley A. (2005). A Physiologically-Based Early Warning Score for Ward Patients: The Association between Score and Outcome. Anaesthesia.

[2] Subbe C.P., Davies R.G., Williams E., Rutherford P., Gemmell L. (2003). Effect of Introducing the Modified Early Warning Score on Clinical Outcomes, Cardio-Pulmonary Arrests and Intensive Care Utilisation in Acute Medical Admissions. Anaesthesia.

[3] Howell M.D., Donnino M.W., Talmor D., Clardy P., Ngo L., Shapiro N.I. (2007). Performance of Severity of Illness Scoring Systems in Emergency Department Patients with Infection. Acad. Emerg. Med..

[4] Chatterjee N.A., Jensen P.N., Harris A.W., Nguyen D.D., Huang H.D., Cheng R.K., Savla J.J., Larsen T.R., Gomez J.M.D., Du-Fay-de-Lavallaz J.M. (2021). Admission Respiratory Status Predicts Mortality in COVID-19. Influenza Other Respir. Viruses.

[5] Wang J., Yu H., Hua Q., Jing S., Liu Z., Peng X., Cao C., Luo Y. (2020). A Descriptive Study of Random Forest Algorithm for Predicting COVID-19 Patients Outcome. PeerJ.

[6] Chourpiliadis C., Bhardwaj A. (2022). Physiology, Respiratory Rate. StatPearls.

[7] Cretikos M.A., Bellomo R., Hillman K., Chen J., Finfer S., Flabouris A. (2008). Respiratory Rate: The Neglected Vital Sign. Med. J. Aust..

[8] Tarassenko L., Clifton D.A., Pinsky M.R., Hravnak M.T., Woods J.R., Watkinson P.J. (2011). Centile-Based Early Warning Scores Derived from Statistical Distributions of Vital Signs. Resuscitation.

[9] Flenady T., Dwyer T., Applegarth J. (2017). Explaining Transgression in Respiratory Rate Observation Methods in the Emergency Department: A Classic Grounded Theory Analysis. Int. J. Nurs. Stud..

[10] Drummond G.B., Fischer D., Arvind D.K. (2020). Current Clinical Methods of Measurement of Respiratory Rate Give Imprecise Values. ERJ Open Res..

[11] Latten G.H.P., Spek M., Muris J.W.M., Cals J.W.L., Stassen P.M. (2019). Accuracy and Interobserver-Agreement of Respiratory Rate Measurements by Healthcare Professionals, and Its Effect on the Outcomes of Clinical Prediction/Diagnostic Rules. PLoS ONE.

[12] Massaroni C., Nicolò A., Lo Presti D., Sacchetti M., Silvestri S., Schena E. (2019). Contact-Based Methods for Measuring Respiratory Rate. Sensors.

[13] Prigent G., Aminian K., Rodrigues T., Vesin J.-M., Millet G.P., Falbriard M., Meyer F., Paraschiv-Ionescu A. (2021). Indirect Estimation of Breathing Rate from Heart Rate Monitoring System during Running. Sensors.

[14] Witt J.D., Fisher J.R.K.O., Guenette J.A., Cheong K.A., Wilson B.J., Sheel A.W. (2006). Measurement of Exercise Ventilation by a Portable Respiratory Inductive Plethysmograph. Respir. Physiol. Neurobiol..

[15] Kim J.-H., Roberge R., Powell J.B., Shafer A.B., Jon Williams W. (2013). Measurement Accuracy of Heart Rate and Respiratory Rate during Graded Exercise and Sustained Exercise in the Heat Using the Zephyr BioHarness. Int. J. Sports Med..

[16] Liu Y., Zhu S.H., Wang G.H., Ye F., Li P.Z. (2013). Validity and Reliability of Multiparameter Physiological Measurements Recorded by the Equivital LifeMonitor during Activities of Various Intensities. J. Occup. Environ. Hyg..

[17] Lombard W.P. (1937). Proceedings of the American Physiological Society. Am. J. Physiol. Leg. Content..

[18] Wu T., Blazek V., Schmitt H.J., Priezzhev A.V., Oberg P.A. (2000). Photoplethysmography Imaging: A New Noninvasive and Noncontact Method for Mapping of the Dermal Perfusion Changes.

[19] Takano C., Ohta Y. (2007). Heart Rate Measurement Based on a Time-Lapse Image. Med. Eng. Phys..

[20] Verkruysse W., Svaasand L.O., Nelson J.S. (2008). Remote Plethysmographic Imaging Using Ambient Light. Opt. Express.

[21] Allen J. (2007). Photoplethysmography and Its Application in Clinical Physiological Measurement. Physiol. Meas..

[22] Tohma A., Nishikawa M., Hashimoto T., Yamazaki Y., Sun G. (2021). Evaluation of Remote Photoplethysmography Measurement Conditions toward Telemedicine Applications. Sensors.

[23] Sun Y., Papin C., Azorin-Peris V., Kalawsky R., Greenwald S., Hu S. (2012). Use of Ambient Light in Remote Photoplethysmographic Systems: Comparison between a High-Performance Camera and a Low-Cost Webcam. J. Biomed. Opt..

[24] Luo H., Yang D., Barszczyk A., Vempala N., Wei J., Wu S.J., Zheng P.P., Fu G., Lee K., Feng Z.-P. (2019). Smartphone-Based Blood Pressure Measurement Using Transdermal Optical Imaging Technology. Circ. Cardiovasc. Imaging.

[25] Coppetti T., Brauchlin A., Müggler S., Attinger-Toller A., Templin C., Schönrath F., Hellermann J., Lüscher T.F., Biaggi P., Wyss C.A. (2017). Accuracy of Smartphone Apps for Heart Rate Measurement. Eur. J. Prev. Cardiolog..

[26] Pham C., Poorzargar K., Nagappa M., Saripella A., Parotto M., Englesakis M., Lee K., Chung F. (2022). Effectiveness of Consumer-Grade Contactless Vital Signs Monitors: A Systematic Review and Meta-Analysis. J. Clin. Monit. Comput..

[27] Jorge J., Villarroel M., Chaichulee S., Guazzi A., Davis S., Green G., McCormick K., Tarassenko L. Non-Contact Monitoring of Respiration in the Neonatal Intensive Care Unit. Proceedings of the 2017 12th IEEE International Conference on Automatic Face & Gesture Recognition (FG 2017).

[28] Couderc J.-P., Kyal S., Mestha L.K., Xu B., Peterson D.R., Xia X., Hall B. (2015). Detection of Atrial Fibrillation Using Contactless Facial Video Monitoring. Heart Rhythm.

[29] Villarroel M., Jorge J., Pugh C., Tarassenko L. Non-Contact Vital Sign Monitoring in the Clinic. Proceedings of the 2017 12th IEEE International Conference on Automatic Face & Gesture Recognition (FG 2017).

[30] Allado E., Poussel M., Moussu A., Saunier V., Bernard Y., Albuisson E., Chenuel B. (2021). Innovative Measurement of Routine Physiological Variables (Heart Rate, Respiratory Rate and Oxygen Saturation) Using a Remote Photoplethysmography Imaging System: A Prospective Comparative Trial Protocol. BMJ Open.

[31] Fitzpatrick T.B. (1988). The Validity and Practicality of Sun-Reactive Skin Types I through VI. Arch. Dermatol..

[32] Koo T.K., Li M.Y. (2016). A Guideline of Selecting and Reporting Intraclass Correlation Coefficients for Reliability Research. J. Chiropr. Med..

[33] Pai A., Veeraraghavan A., Sabharwal A. (2021). HRVCam: Robust Camera-Based Measurement of Heart Rate Variability. J. Biomed. Opt..

[34] Yue H., Li X., Cai K., Chen H., Liang S., Wang T., Huang W. (2020). Non-Contact Heart Rate Detection by Combining Empirical Mode Decomposition and Permutation Entropy under Non-Cooperative Face Shake. Neurocomputing.

[35] Al-Naji A., Chahl J., Lee S.-H. (2019). Cardiopulmonary Signal Acquisition from Different Regions Using Video Imaging Analysis. Comput. Methods Biomech. Biomed. Eng. Imaging Vis..

[36] Wei B., He X., Zhang C., Wu X. (2017). Non-Contact, Synchronous Dynamic Measurement of Respiratory Rate and Heart Rate Based on Dual Sensitive Regions. BioMed Eng. OnLine.

[37] Sanyal S., Nundy K.K. (2018). Algorithms for Monitoring Heart Rate and Respiratory Rate from the Video of a User’s Face. IEEE J. Transl. Eng. Health Med..

