# Supplementary material for: Remote Photoplethysmography Is an Accurate Method to Remotely Measure Respiratory Rate: A Hospital-Based Trial
Source: J Clin Med. 2022 Jun 24;11(13):3647. doi: 10.3390/jcm11133647 (PMC9267568; doi:10.3390/jcm11133647)
Supplement: Supplementary file 1 [file jcm-11-03647-s001.zip › jcm-1741235-supplementary.pdf]

Supplementary Table S1. Intra-system concordance between rPPGc as a function of time

Time	30 seconds	60 seconds	120 seconds
30s			
60s	0.976 [0.973-0.979]		
120s	0.963 [0.959-0.968]	0.986 [0.984-0.988]	
